# Massage

**Published:** 1881-10

**Authors:** James I. Tucker

**Affiliations:** Chicago


### ARTICLE VII.

MASSAGE. By JAMES I. TUCKER, A.M., M.D. Chicago.

Inasmuch as massage is attracting considerable attention now-a-days, and is thought by many to be a notion of modern times, it occurred to me that the following medico-historical data * might be no less interesting to your other readers than they have been to me.

In India the massage method was in vogue at least two thousand years ago, for a Greek historian, who was in that country 300 years B. C., related that at that time there existed among the Brahmins an order of physicians whose treatment consisted in regulating diet, enjoining abstemiousness and outward manipulations. At the present time, in the valley of the Ganges, there is an order of Brahmins, whose method of treating disease is a hygi-

* See Gartenlaube Heft 14, 1881, p. 445.

enic rubbing and shampooing after the bath. The patient lies outstretched upon a lounge, and the Brahmin kneads his limbs as if they were dough. Then he strokes the body lightly with the palms of his hands, perfumes and anoints him, and concludes by bending and stretching the neck and joints of fingers and toes.

The same method is in vogue in the Orient, especially among the Turks. The beneficial effects of massage were likewise known to the ancient Greeks and Romans, for after their bath they had their slaves rub, knead and anoint them, and Hippocrates considered it important that the physician, among other things necessary to the mastery of his art, should understand massage. Asclepiades, who lived one hundred years before Christ, was likewise an advocate of this method of healing.

It was a Swede named Pehr Hewrik Ling, who, while founding a system of gymnastics upon purely anatomico-physiological principles, introduced passive motion. This occurred in the second decade of this century, and the students of Ling brought the massage method to great perfection. For a long time all kinds of passive motion, like stroking, patting, pounding, kneading, rubbing, pinching, stretching, etc., have been applied with great success.

Of late a Hollander named Metzger, who has achieved brilliant success by the help of massage, attracts to himself both the learned and the laity, some to place themselves under treatment, and some to learn his method.

His method consists principally of four kinds of manipulation, to wit:

1. *Effleuvage:* slow and gentle stroking with the palms of the hands.

2. *Massage à friction:* forcible stroking and rotary rubbing movement in alternation, either with one hand or with both hands.

3. *Pétrissage:* kneading.

4. *Tapatement:* pounding, beating and slapping with the flat hand or its edge, and with the closed fist.

So important an adjunct to the medical art as massage, that rests upon a scientific basis, and endorsed by so many German, French, English and American physicians, including Esmarsh, Charcot and Mitchell, should not be overlooked by any of the members of our profession.

